# An Adaptive Multi-Mode Navigation Method with Intelligent Virtual Sensor Based on Long Short-Term Memory in GNSS Restricted Environment

**DOI:** 10.3390/s23084076

**Published:** 2023-04-18

**Authors:** Rong Wang, Yu Rui, Jingxin Zhao, Zhi Xiong, Jianye Liu

**Affiliations:** Navigation Research Center, College of Automation Engineering, Nanjing University of Aeronautics and Astronautics, Nanjing 211106, China

**Keywords:** INS/GNSS integrated navigation system, GNSS restricted environment, long short-term memory, back propagation neural networks, fireworks optimization algorithm

## Abstract

Aiming at the problem of fast divergence of pure inertial navigation system without correction under the condition of GNSS restricted environment, this paper proposes a multi-mode navigation method with an intelligent virtual sensor based on long short-term memory (LSTM). The training mode, predicting mode, and validation mode for the intelligent virtual sensor are designed. The modes are switching flexibly according to GNSS rejecting situation and the status of the LSTM network of the intelligent virtual sensor. Then the inertial navigation system (INS) is corrected, and the availability of the LSTM network is also maintained. Meanwhile, the fireworks algorithm is adopted to optimize the learning rate and the number of hidden layers of LSTM hyperparameters to improve the estimation performance. The simulation results show that the proposed method can maintain the prediction accuracy of the intelligent virtual sensor online and shorten the training time according to the performance requirements adaptively. Under small sample conditions, the training efficiency and availability ratio of the proposed intelligent virtual sensor are improved significantly more than the neural network (BP) as well as the conventional LSTM network, improving the navigation performance in GNSS restricted environment effectively and efficiently.

## 1. Introduction

The navigation of unmanned aerial vehicle (UAV) mainly relies on the integrated navigation system composed of an inertial navigation system (INS) and global navigation satellite system (GNSS), which has the characteristics of high navigation accuracy and continuous all-weather operation. However, when the UAV is under interference or deception environment, sufficient GNSS signals cannot be received to ensure continuous and stable positioning in the whole task process. Then in the case of the GNSS restriction, INS positioning errors will accumulate rapidly with time. Therefore, the study of suppressing fast divergence of inertial navigation positioning errors when the GNSS signal is unavailable has important practical application value.

At present, most of the GNSS/INS integrated navigation systems operate based on the Kalman filter. However, the update of the Kalman filter in the GNSS/INS integrated navigation system relies on the measurement of the GNSS receiver and will lose effectiveness under GNSS unavailable environment. The development of artificial neural networks helps solve this problem. When the INS error compensation cannot be carried out due to signal interruption of the auxiliary sensor in integrated navigation, the neural network is used to improve the accuracy of integrated navigation [1]. XU Aigong proposed an integrated navigation algorithm assisted by a radial basis neural network (RBF) [2]. The trained RBF neural network was used to predict and correct the position error of INS. Doostdar proposed a recursive fuzzy wavelet neural network (RFWNN) to assist the integrated navigation system. When GNSS is available, the position, velocity, and error results of SINS are used to assist SINS and the velocity; the position of SINS and data provided by GNSS are used to train RFWNN. When the GNSS is interrupted, RFWNN is used to provide accurate position and velocity error values as the observation values of the Kalman filter, to reduce the error accumulation when SINS work independently [3]. Mou R proposed a GNSS/INS integrated navigation system model assisted by a BP neural network when GNSS signals were missing and used a BP neural network to replace the Kalman filter to complete the error compensation of INS data and guarantee the overall accuracy of the system [4]. Nie Jianliang used the adaptive Kalman filter for training the link weight of the hidden lay and output lay to improve the capability of the BP neural network. By comparison, the precision and generalization ability of BP based on adaptive Kalman filtering is higher than those of the traditional BP neural network [5]. Chong, Y uses geomagnetic suitability areas (GSA) to modify the initial weights of BP neural networks to optimize BP [6]. Zhang, L adopted an artificial bee colony (ABC) algorithm to select visible satellites for positioning in a multi-constellation satellite navigation system with the minimum geometric dilution of precision (GDOP) value achieved [7]. However, almost all the above networks, such as RFWNN and BP, belong to static neural networks. Their main disadvantage is that these neural networks cannot store more dynamic information about the aircraft at the previous time [8]. In practical applications, after the outage of GNSS, inertial navigation errors will accumulate over time, and it is difficult for BP neural network to analyze the timing sequence characteristics of inertial navigation errors. Moreover, BP neural network has problems such as slow convergence speed and easy to fall into local minimum when processing large quantities of data.

To solve the problem that traditional neural networks cannot process time series, this paper uses a long short-term memory network (LSTM) to assist the inertial navigation system when GNSS is unavailable. LSTM is an excellent variant type of RNN network and has the property of saving the state of previous data, so it is suitable for dealing with time series-sensitive problems and tasks [9]. At the same time, LSTM solves the Vanishing Gradient problem caused by a gradual reduction in the process of gradient backpropagation [10,11,12].

In the traditional neural network-assisted navigation method, there are generally only two modes: training mode and predicting mode. The traditional neural network-assisted navigation method cannot update the neural network at a time when the carrier motion state changes, so the prediction accuracy is difficult to meet the current navigation needs. Moreover, the hyperparameters of the neural network such as the learning rate and the number of hidden layers are usually set in advance, which is difficult to find the optimal parameters in the application.

The LSTM network in this paper can replace the original positioning function of GNSS when GNSS is unavailable, which is similar to a virtual sensor. Therefore, this paper proposes an adaptive multi-mode navigation method with an intelligent virtual sensor based on LSTM in a GNSS-restricted environment. Compared with the traditional neural network-assisted navigation method, we add a new validation mode. The modes are switching flexibly according to GNSS rejecting situation and the status of the LSTM network of the intelligent virtual sensor. Then the inertial navigation system (INS) is corrected and the availability of the LSTM network is also maintained. Meanwhile, the fireworks algorithm (FWA) is adopted to optimize the learning rate and the number of hidden layers of LSTM hyperparameters to improve the estimation performance.

## 2. Scheme Design for Loose Coupling Navigation under GNSS Blocking

### 2.1. Traditional INS/GNSS Loose Coupling Navigation Scheme

**Figure 1:**
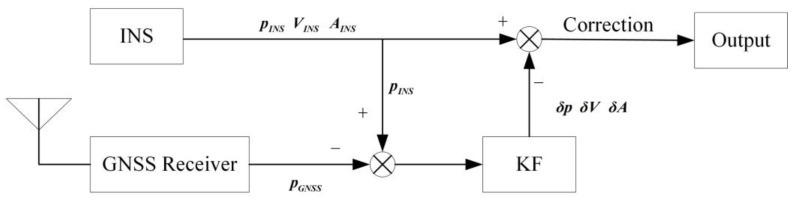
Traditional GNSS/INS loose coupling navigation system.

A loose coupling navigation system is manifested in the INS assisted by the GNSS receiver with a Kalman filter [13], as illustrated in Figure 1.

In Figure 1, $p_{INS}$, $v_{INS}$, $a_{INS}$ represent the position, velocity and attitude provided by INS, respectively. $p_{GNSS}$ represents the position provided by the GNSS receiver [14]. The difference between the position provided by the GNSS receiver and INS is used as the observation of the Kalman filter, and then the error of INS is estimated by the Kalman filter and used in compensating for the errors in the INS. The update of the Kalman filter in the GNSS/INS loose coupling navigation system relies on the measurement of the GNSS receiver. The Kalman filter could not work when the GNSS signal is an outage, and the INS error could not be estimated and compensated. Then the navigation error will diverge rapidly according to the cumulative effect of errors in INS [15,16,17].

### 2.2. Proposed Loose Coupling Navigation Scheme with Intelligent Virtual Sensor

**Figure 2:**
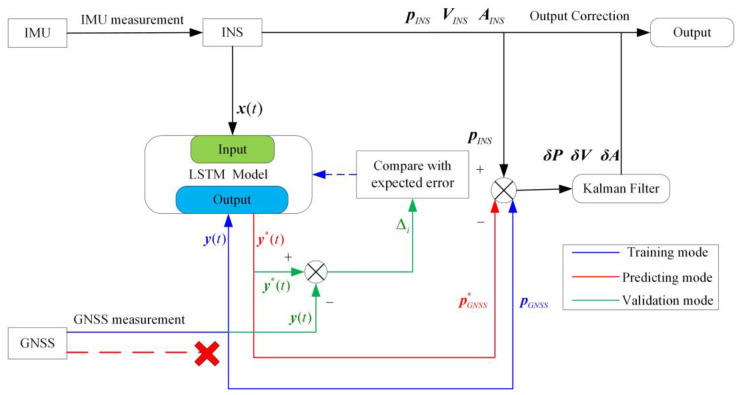
Structure Diagram of Loose Coupling Navigation Scheme with Intelligent Virtual Sensor.

To solve the problem that the INS error compensation cannot be carried out due to signal interruption of the GNSS receiver in loose coupling navigation, the neural network is introduced. Figure 2 shows the structure diagram of the loose coupling navigation scheme with the intelligent virtual sensor.

In Figure 2, there are three modes designed for the virtual sensor: the training mode, the predicting mode, and the validation mode. The data sets x(t) of the INS measurements will be fed into the LSTM neural network in either mode, and the selection of x(t) will be covered in detail in Section 3.2. The features of the three modes are as follows:

(1) Training mode

The blue lines indicate the direction of signal flow in training mode used in the GNSS available environment, in which the position increment obtained by the processing of GNSS measurement information is used as the training output of the LSTM network.

The system states vector X is composed of the error of INS.

(1) $$\begin{array}{l} X=[\varphi_{E}\;\varphi_{N}\;\varphi_{U}\;\delta v_{E}\;\delta v_{N}\;\delta v_{U}\;\delta L\;\delta \lambda \;\delta h\;\\ \;\;\varepsilon_{bx}\;\varepsilon_{by}\;\varepsilon_{bz}\;\varepsilon_{rx}\;\varepsilon_{ry}\;\varepsilon_{rz}\;\nabla_{x}\;\nabla_{y}\;\nabla_{z}{]}^{T} \end{array}$$

where φ is the inertial navigation platform error angles in the n-frame. δv are the velocity errors in the n-frame. δL, δλ, and δh, respectively, represent the position errors of latitude, longitude, and height. $\varepsilon_{bx}$, $\varepsilon_{by}$, $\varepsilon_{bz}$, $\varepsilon_{rx}$, $\varepsilon_{ry}$, $\varepsilon_{rz}$, $\nabla_{x}$, $\nabla_{y}$ and $\nabla_{z}$ represent the accelerometer biases and gyroscope biases in three axes in the b-frame.

The error model of the INS is selected as the state equation:

(2) $$X_{k}=\Phi_{k|k-1}X_{k-1}+\Gamma_{k-1}W_{k-1}$$

where $\Phi_{k,k-1}$ is the state transfer matrix of the system from epoch k−1 to epoch k, $\Gamma_{k-1}$ is the system noise matrix, $W_{k-1}$ is the system noise at epoch k−1, and $Q_{k}$ is the covariance matrices of system noise.

To facilitate the design of the integration algorithm, longitude and latitude are often used as the observation quantity in integrated navigation. In the loose coupling navigation adopted in this paper, the observed position is the difference between the longitude, latitude, and altitude information given by the inertial navigation system and the corresponding information given by the GNSS receiver. When GNSS is available, the difference between the position provided by the INS and provided by the GNSS receiver is defined as the observation for filtering, and the measurement equation is:

(3) $$\begin{array}{cc} Z_{\mathrm{Measure}\;k} & =H_{k}X_{k}+V_{k}\\ & =\left[\begin{array}{l} (L_{INS}-L_{GNSS})R_{M}\\ (\lambda_{INS}-\lambda_{GNSS})R_{N}\cos L\\ h_{INS}-h_{GNSS} \end{array}\right] \end{array}$$

where $R_{M}$ represents the radius of curvature of the meridian circle, $R_{N}$ represents the radius of curvature of the unitary circle, $\left(L_{GNSS},\lambda_{GNSS},h_{GNSS}\right)$ and $\left(L_{INS},\lambda_{INS},h_{INS}\right)$ represent longitude, latitude, and altitude position information measured by GNSS receiver or INS, respectively.

(4) $$Z_{k}=H_{k}X_{k}+V_{k}$$

where $V_{k}$ is the measurement noise at epoch k, and $R_{k}$ are the covariance matrices of the observation respectively.

The state one-step prediction equation is

(5) $${\hat{X}}_{k|k-1}=\Phi_{k,k-1}{\hat{X}}_{k-1|k-1}$$

where ${\hat{X}}_{k-1}$ is the estimate states, and ${\hat{X}}_{k|k-1}$ is a one-step prediction of the states.

The one-step prediction mean square error equation of KF is

(6) $$P_{k|k-1}=\Phi_{k,k-1}P_{k-1}\Phi_{k,k-1}^{T}+\Gamma_{k-1}Q_{k-1}\Gamma_{k-1}^{T}$$

The filter gain equation is

(7) $$K_{k}=P_{k|k-1}H_{k}^{T}{(H_{k}P_{k|k-1}H_{k}^{T}+R_{k})}^{-1}$$

The state valuation equation is

(8) $${\hat{X}}_{k|k}={\hat{X}}_{k|k-1}+K_{k}(Z_{k}-H_{k}{\hat{X}}_{k|k-1})$$

The estimated mean square error equation is

(9) $$P_{k|k}=(I-K_{k}H_{k})P_{k|k-1}$$

$K_{k}$ is the Kalman gain matrix, $P_{k}$ is the estimated covariance matrix, and $P_{k|k-1}$ is the covariance matrix for estimating ${\hat{X}}_{k|k-1}$. Then the INS error states ${\hat{X}}_{k|k}$ estimated according to GNSS measurements is fed back to the INS for error compensation.

(2) Predicting mode

The red lines indicate the direction of signal flow in predicting mode used in the GNSS unavailable environment, in which the position increment is predicted by the LSTM network. Then the pseudo-localization solution of the Virtual Sensor, denoted as $Z_{Virtual}$, is calculated from the predicted position increment and then input into the measurement update of the Kalman filtering to estimate and compensate the errors of INS. Then the measurement equation is defined as:

(10) $$\begin{array}{cc} Z_{\mathrm{Virtual}\;k} & =H_{k}X_{k}+V_{k}\\ & =\left[\begin{array}{c} (L_{INS}-L_{Virtual})R_{M}\\ (\lambda_{INS}-\lambda_{Virtual})R_{N}\cos L\\ h_{INS}-h_{Virtual} \end{array}\right] \end{array}$$

where $\left(L_{Virtual},\lambda_{Virtual},h_{Virtual}\right)$ represent longitude, latitude, and altitude position obtained from virtual sensor with LSTM network prediction. The construction of the virtual sensor will be discussed in Section 4.

(3) Predicting mode

The green lines indicate the direction of signal flow in validation mode used to evaluate the status of the virtual sensor, in which the pseudo-localization solution obtained according to the LSTM network prediction is compared with the position information after the GNSS signal recovery. When the error exceeds the expected threshold, the LSTM network will be trained again to maintain the performance of the virtual sensor.

It is important to note that it is critical to monitor and maintain the effectiveness of the virtual sensors. On the one hand, it is difficult to guarantee that a virtual sensor built on the basis of data training for one scenario can accurately predict and simulate the measurement state in another, due to the dynamic environment and measurement conditions that change constantly during the carrier operation. Therefore, the state of the virtual sensor needs to be evaluated using a validation model after GNSS recovery, and a new round of training needs to be initiated accordingly. On the other hand, the training of virtual sensors can also take up a significant amount of system resources and delay the time available for prediction. Therefore, when the virtual sensor is monitored to have reached the required training degree, it needs to be shifted to a prediction-ready state in time to improve training efficiency.

## 3. Optimal Pseudo-Positioning Based on LSTM for GNSS Blocking

### 3.1. Structure of the LSTM Network

**Figure 3:**
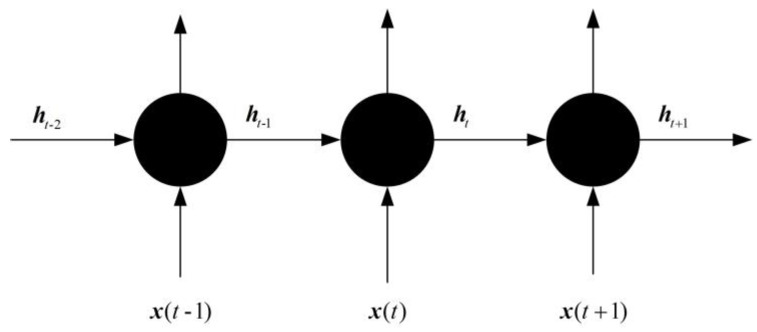
Traditional RNN structure diagram.

**Figure 4:**
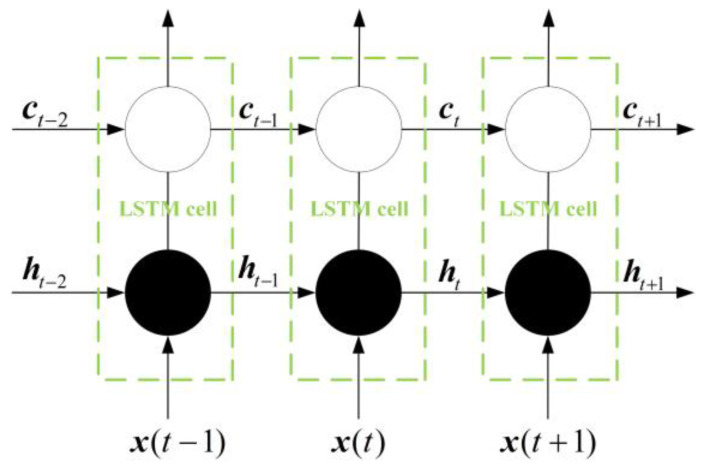
LSTM structure diagram.

The design idea of the LSTM network is relatively simple. The hidden layer in the original RNN has only one state h, which is sensitive to short-term input. Now a state c called a cell state is added to save the long-term state [18]. x(t) represents the training input of the neural network at the time t. The network before and after adding the cell state is compared, as shown in Figure 3 and Figure 4.

**Figure 5:**
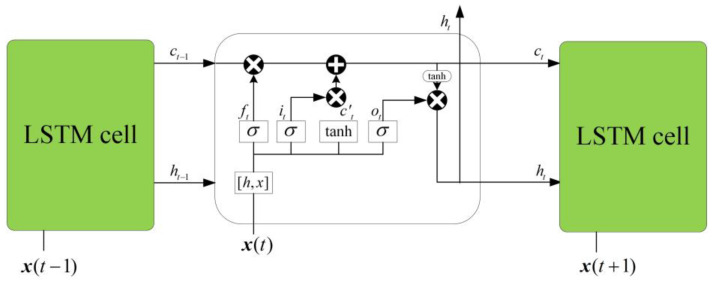
Schematic diagram of LSTM internal structure.

LSTM designed a gated structure to control the retention and discarding of information. The LSTM has three gates. Figure 5 shows the structure of the LSTM: forget gate, input gate, and output gate.

LSTM cyclic networks do not simply apply an element-by-element nonlinearity to affine transformations of input and cyclic elements. $U_{t}$ represents the forget gate, in which the information output of the node at the last time can be limited. $i_{t}$ represents the input gate, in which the new input information can be selectively obtained, and $o_{t}$ represents the output gate, in which the output at the current time can be transmitted according to the new input information and the previous time [19]. Figure 5 shows the common LSTM internal structure.

In Figure 5, $f_{t}$ represents the output of the forget gate, and the Sigmoid activation function is adopted to map the output to the interval [0, 1]. When the cell state $c_{t-1}$ passes through the oblivion gate at the previous time, the result will be multiplied to $f_{t}$. Obviously, the information with a multiplier of 0 is all discarded, and the information with a multiplier of 1 is retained. This determines how much of the previous cell state $c_{t-1}$ can enter the current state $c_{t}$.

The formula of the forgot gate is

(11) $$f_{t}=\sigma (h_{t-1}∙\;W_{f}+x(t-1)∙\;U_{f}+b_{f})$$

where σ represents the sigmoid activation function, and $h_{t-1}$ represents the hidden layer state at the last moment. x(t−1) represents the input at the current time. $W_{f}$ and $U_{f}$ represent the parameter matrix, and $b_{f}$ represents the parameter vector.

The input gate $i_{t}$ determines what input information is retained. The input information includes two parts: the input at the current time and the output of the hidden layer at the previous time and is stored in the immediate cell state ${\tilde{c}}_{t}$. The input gate still uses the Sigmoid activation function to map the output to the interval [0, 1]. ${\tilde{c}}_{t}$ filters information through the input gate.

The formula of the input gate is

(12) $$i_{t}=\sigma \;(h_{t-1}∙W_{i}+x(t)∙U_{i}+b_{i})$$

The formula of the instant cell state is

(13) $${\tilde{c}}_{t}=\tanh(h_{t-1}∙W_{c}+x(t)∙U_{c}+b_{c})$$

The information retained at the previous time, plus the information retained by the current recipient, forms the cell state $c_{t}$ at the present time.

The formula of the current cell state is

(14) $$c_{t}=f_{t}\circ c_{t-1}+i_{t}\circ {\tilde{c}}_{t}$$

The output gate determines what information is in $h_{t-1}$, and $x_{t}$ will be output.

The formula of the output gate is

(15) $$h_{t}=o_{t}\circ \tanh(c_{t})$$

where ∙ represents the matrix product and ∘ represents the element product [20,21].

### 3.2. Design the LSTM Network for Pseudo-Positioning

When GNSS is available, the output data of the gyroscope and accelerometer are calculated and converted to obtain the carrier angular velocity $\omega_{IMU}$ and carrier acceleration $a_{IMU}$, and the carrier angular velocity information and carrier acceleration information output by the inertial navigation system during the flight of the aircraft are stored. According to the angular velocity and acceleration information of the carrier output by the inertial navigation system, the difference between the angular and velocity information output by the inertial navigation system at the time $t_{k-1}$ to $t_{k}$ is made to obtain the angular and velocity increment of the aircraft output by the inertial navigation system. The calculation formula is as follows:

(16) $$\left\{\begin{array}{l} \Delta \Omega_{IMU}={\left[\Delta \Omega_{X}^{t}\;\;\Delta \Omega_{Y}^{t}\;\;\Delta \Omega_{Z}^{t}\right]}^{T}=\int (\omega_{IMU_{t_{k}}}-\omega_{IMU_{t_{k-1}}})\cdot dt\\ \Delta v_{IMU}={\left[\Delta v_{X}^{t}\;\;\Delta v_{Y}^{t}\;\;\right]}^{T}=\int (a_{IMU_{t_{k}}}-a_{IMU_{t_{k-1}}})\cdot dt \end{array}\right.$$

where $\Delta \Omega_{IMU}$ represents the angular increment of the aircraft system, $\Delta v_{IMU}$ represents the velocity increment of the aircraft system, $\omega_{IMU_{t_{k}}}$ represents the angular velocity of the carrier measured by gyroscope at the time $t_{k}$ and converted, $\omega_{IMU_{t_{k-1}}}$ represents the angular velocity of the carrier measured by gyroscope at the time $t_{k-1}$ and converted, $a_{IMU_{t_{k}}}$ represents the acceleration of the carrier measured by the accelerometer at the time $t_{k}$ and converted, $a_{IMU_{t_{k-1}}}$ represents the acceleration of the carrier measured by the accelerometer at the time $t_{k-1}$ and converted, $\Delta \Omega_{X}^{t}$ represents the carrier X axis Angle increment from time $t_{k-1}$ to time $t_{k}$, $\Delta \Omega_{Y}^{t}$ represents the carrier Y axis Angle increment from time to time, $\Delta \Omega_{Z}^{t}$ is the carrier Z axis Angle increment from time $t_{k-1}$ to time $t_{k}$, $\Delta v_{X}^{t}$ represents the carrier X axis velocity increment from time $t_{k-1}$ to time $t_{k}$, and $\Delta v_{Y}^{t}$ represents the carrier Y axis velocity increment from time $t_{k-1}$ to time $t_{k}$.

The angle and velocity increment data of the output of the inertial navigation system when GNSS is effective were taken as the training input sequence, normalized processing was carried out, and the standardized input data x(t) for training was obtained. The expression is as follows:

(17) $$x(t)={\left[\Delta V_{X}^{t}\;\Delta V_{Y}^{t}\;\Delta \Omega_{X}^{t}\;\Delta \Omega_{Y}^{t}\;\Delta \Omega_{Z}^{t}\;\right]}^{T}$$

The longitude position and latitude position of the aircraft measured by GNSS were obtained, and the difference of the position information output by GNSS from time $t_{k-1}$ to time $t_{k}$ was made to obtain the longitude and latitude position increment of the aircraft:

(18) $$\left\{\begin{array}{l} \Delta P_{L}^{t}=L_{t_{k}}-L_{t_{k-1}}\\ \Delta P_{\lambda }^{t}=\lambda_{t_{k}}-\lambda_{t_{k-1}} \end{array}\right.$$

where $\Delta P_{L}^{t}$ represents the longitude position increment, $\Delta P_{\lambda }^{t}$ represents the latitude position increment, $L_{t_{k}}$ represents the longitude position of the aircraft at the time $t_{k}$, $L_{t_{k-1}}$ represents the longitude position of the aircraft at the time $t_{k-1}$, $\lambda_{t_{k}}$ represents the latitude position of the aircraft at the time $t_{k}$, and $\lambda_{t_{k-1}}$ represents the latitude position of the aircraft at the time $t_{k-1}$.

The increment of the longitude and latitude position of the aircraft when GNSS is available is taken as the training output sequence, normalized processing was carried out, and the standardized output data y(t) for training is obtained, and the expression is:

(19) $$y(t)=\left[\Delta P_{L}^{t}\;\Delta P_{\lambda }^{t}\right]$$

**Figure 6:**
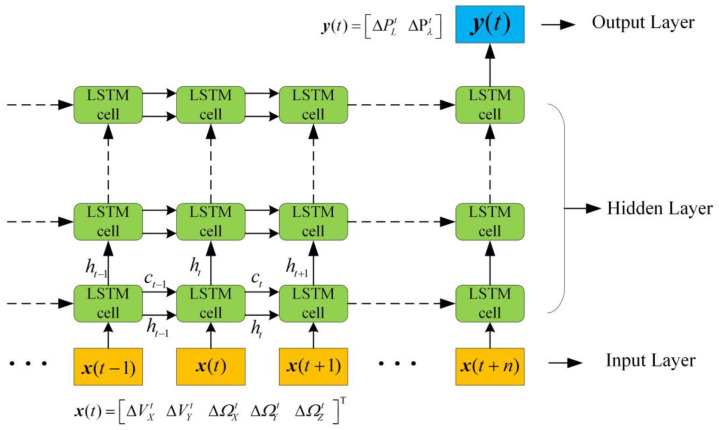
LSTM block diagram for constructing virtual sensor.

LSTM block diagram for constructing a virtual sensor is shown in Figure 6, yellow represents the input layer, $x(t)={\left[\Delta V_{X}^{t}\;\Delta V_{Y}^{t}\;\Delta \Omega_{X}^{t}\;\Delta \Omega_{Y}^{t}\;\Delta \Omega_{Z}^{t}\;\right]}^{T}$, green represents the hidden layer, and blue represents the output layer [22], the last state of the last layer of the network is selected as the output after the activation function.

In this way, LSTM solves the problem that traditional RNN is difficult to deal with long-distance dependence, makes the neural network better able to train and solve the task with time sequence information, and greatly expands the application scope of the neural network [23].

### 3.3. Optimization Pseudo-Positioning Based on LSTM in GNSS Blocking

In the process of designing the LSTM network architecture, there are two important configurable hyperparameters that need to be adjusted, which are the number of hidden layers of the LSTM cell and the learning rate. Setting too many hidden layers and time steps will, as a result, end in the weight update being too large, and the performance of the LSTM model (such as its loss on the training data set) will oscillate during the training period, leading to longer training time to make the algorithm converge. Moreover, it may lead to an overfitting problem, and the results of network training will always be difficult to reach optimal. Therefore, the concept of learning rate attenuation is introduced in this paper, that is, at the initial stage of model training, a large learning rate is used to optimize the model. With the increase in the number of iterations, the learning rate will gradually decrease to ensure that the model will not have too much fluctuation in the later stage of training, to get closer to the optimal solution [24,25].

At present, LSTM uses manual adjustment of hyperparameters for inertial navigation correction, which is inefficient and difficult to find the optimal parameters. Based on this, this paper introduces the fireworks algorithm to optimize the number of LSTM hidden layers and learning rate. FWA algorithm is a new group intelligent optimization algorithm that mimics a fireworks explosion. It simulates a fireworks explosion scene, establishes a relative mathematical model, introduces a random variation strategy and selection strategy, and proposes a global optimization method based on the explosion.

FWA algorithm consists of an explosion operator, mutation operator, mapping rule, and selection strategy. The explosion operator of FWA is designed to differentiate fireworks with different fitness values, that is to consider both local accurate search and global efficient search [26]. The expression of firework $z_{i}$ in this paper is

(20) $$z_{i}=[N,\eta ]$$

where N represents the number of hidden layers, and η represents the learning rate.

The calculation formulas of explosive spark quantity $S_{i}$ and explosion radius $A_{i}$ of firework $z_{i}$ in FWA are

(21) $$S_{i}=M\times \frac{y_{\max}-f(z_{i})+\varepsilon }{\sum_{i=1}^{n}\left(y_{\max}-f(z_{i})\right)+\varepsilon }$$

(22) $$A_{i}=\hat{A}\times \frac{f(z_{i})-y_{\min}+\varepsilon }{\sum_{i=1}^{n}\left(f(z_{i})-y_{\min}\right)+\varepsilon }$$

where $y_{\min}$ is the minimum fitness of the current firework population, and $y_{\max}$ is the maximum fitness of the current firework population. M is a constant that adjusts the number of sparks produced by the explosion. $f(z_{i})$ represents LSTM training architecture. $\hat{A}$ is also a constant that adjusts the radius of the explosion, ε is the minimum amount of the machine, so that there will be no division by zero operation in the calculation process.

To control the amount of computation not too large, and to achieve the purpose of the global efficient search, the number of sparks generated ${\overset{\wedge }{S}}_{i}$ is limited:

(23) $${\hat{S}}_{i}=\left\{\begin{array}{l} round(\mathrm{aM}),\;\;\;S_{i}<\mathrm{aM}\\ round(\mathrm{bM}),\;\;\;S_{i}>\mathrm{bM},\;a<b<1\\ round(S_{i}) \end{array}\right.$$

where a, b are all constant, and round(∙) is the rounded integer function.

To improve population diversity, mutation operators were added to FWA to generate mutation sparks. For the dimension k selected by one of the firework $z_{i}$, the Gaussian mutation operation is:

(24) $${\hat{z}}_{ik}=z_{ik}\times e$$

where e follows a Gaussian distribution with a mean of 1 and a variance of 1.

To prevent the spark generated by the explosion operator and mutation operator from exceeding the boundary of the solution range, the mapping rule % is used to transform ${\hat{z}}_{ik}$ into the solution range. The mapping rule is

(25) $${\hat{z}}_{ik}={\hat{z}}_{\min,k}+|{\hat{z}}_{ik}|\%({\hat{z}}_{\max,k}-{\hat{z}}_{\min,k})$$

where $z_{\max,k}$, $z_{\min,k}$ represent the boundaries of the solution space on dimension k.

To make the fireworks in the optimal position in the population inherit, the selection strategy is adopted. The selection strategy adopted in this paper is to sort each spark $z_{i}$ according to the fitness, select the fireworks individual with the least fitness, and deterministically select the next generation as fireworks. The fitness of fireworks designed in this paper is the average value of LSTM neural network prediction error under this parameter. The formula for calculating the selection probability $R_{P}(z_{i})$ of the remaining fireworks $z_{i}$ is:

(26) $$R_{P}(z_{i})=\frac{R(z_{i})}{\sum_{z_{i}\in K}R(z_{i})}$$

(27) $$R(z_{i})=\sum_{z_{i}\in K}\left\|z_{i}-z_{j}\right\|$$

where $R(z_{i})$ represents the sum of the distance between the current spark and all sparks except itself, and K is the set of all the remaining fireworks.

The smaller the $R_{P}(z_{i})$ is, the better the performance of the fireworks $z_{i}$ as the parameter of the LSTM network. The right amount of fireworks is selected according to the $R_{P}(z_{i})$ value. Finally, all the selected fireworks are sorted again for fitness, and the fireworks with the least fitness are used as the parameter of the LSTM network [27].

LSTM parameters optimized by the fireworks algorithm can provide more accurate predicted values and avoid the overfitting problem of the LSTM neural network. However, the addition of optimization will also increase the system’s uptime.

## 4. Intelligent Integrated Navigation Algorithm with Virtual Sensor

### 4.1. System Modes of Virtual Sensor

#### 4.1.1. Training Mode

In the training mode, the training output of the LSTM network is the position increment obtained by GNSS output after processing. The higher the GNSS accuracy, the better the training effect of the LSTM network will be natural. However, it is necessary to judge whether the position accuracy of the current GNSS is suitable for training the LSTM network, and the judgment condition is whether the prediction effect of the neural network trained with the output data of the current GNSS is better than that of the divergent inertial navigation.

The LSTM network model is built to learn the current training input and training output. The input of the LSTM network is $X\in \left\{x(t)|t_{k-1}<t<t_{k}\right\}$, and the training output of the LSTM network is $Y\in \left\{y(t)|t_{k-1}<t<t_{k}\right\}$, which is elaborated in Section 3.2. When the training error is greater than the set threshold, the parameters of the LSTM network are optimized by the FWA optimization method, and the number of hidden layers and learning rate in LSTM network parameters are determined and adjusted adaptively. The adjustment method has been covered in detail in Section 3.3. After the LSTM training error becomes stable, the training effect is verified through the validation mode. If the validation error is lower than the threshold, the training is ended. If it is greater than the threshold, the training continues.

#### 4.1.2. Predicting Mode

When GNSS is unavailable, the system changes from training mode to predicting mode.

The angle and velocity increment $X\in \left\{x(t)|t_{k-1}<t<t_{k}\right\}$ of the inertial navigation system output when GNSS is unavailable are taught by the trained LSTM network, and the prediction array of the LSTM network is output and de-standardized to obtain the position increment array $Y^{*}\in \left\{y^{*}(t)|t_{k-1}<t<t_{k}\right\}$ of LSTM network prediction, and the expression of $y^{*}(t)$ is:

(28) $$y^{*}(t)=\left[\Delta P_{L}^{t*}\;\;\;\Delta P_{\lambda }^{t*}\right]$$

The position of the aircraft at the moment of GNSS signal loss $p_{GNSS(t_{s})}$ is obtained, and the position increment pseudo-observation information $y^{*}(t)$ predicted by the LSTM network is accumulated to obtain the position pseudo-observation information $p_{GNSS}^{*}$ calculated after GNSS signal loss as follows:

(29) $$p_{GNSS}^{*}=p_{GNSS(t_{s})}+\sum_{k=2}^{m}y^{*}(t)\;\left(t_{k-1}<t<t_{k}\right)$$

where $t_{s}$ is the moment when the signal from GNSS is lost, and m is the prediction duration. In the predicting mode, the prediction frequency of the LSTM network is 1 Hz.

#### 4.1.3. Validation Mode

After the GNSS signal recovers, the system enters the validation mode.

The pseudo position increment predicted by the LSTM network was compared with the position increment measured by the GNSS that recovered to normal, and the difference was compared with the expected error to determine whether the LSTM network needs to continue training. The progress of training was monitored, and the training residuals $\Delta_{i}$ is calculated, with the formulas as follows:

(30) $$\Delta_{i}=\sqrt{\frac{\sum_{k=2}^{u}{\left(y^{*}(t)-y(t)\right)}^{2}}{u}}\;(t_{k-1}<t<t_{k})$$

where $y^{*}(t)$ represents the position increment pseudo-observation information, y(t) represents the position increment information observed by recovered GNSS, and u represents the validation duration. In the validation mode, the prediction frequency of the LSTM network is still 1 Hz.

If $\Delta_{i}$ is greater than the set LSTM network expected error, the system enters the training mode again. Otherwise, the training is stopped.

### 4.2. Data Processing Time Sequence Design

**Figure 7:**
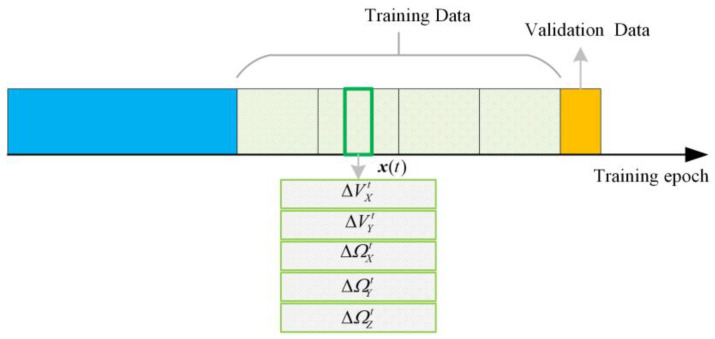
The data structure of the LSTM training mode.

Figure 7 shows the data structure of the LSTM training. Since the movement of the aircraft is dynamic when a state such as manoeuvring occurs, the training data received by the LSTM may not produce reliable prediction results, and it is necessary to continue training the LSTM network, so a verification module needs to be designed. When the training is completed, the validation set sequence of the effective GNSS is used to check the effectiveness of the training and determine whether it is necessary to continue the training.

**Figure 8:**
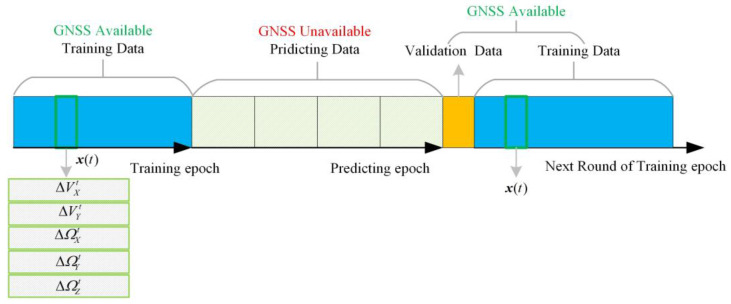
The data structure of the LSTM predicting mode.

Figure 8 shows the data structure of the LSTM prediction. When GNSS signals are unavailable, the sequence vector x(t) at every moment will be sent to the LSTM network as the prediction input. After GNSS signal recovery, LSTM may have a poor predictive effect on the new flight path, so further training is needed. The new training will prolong the operation time of the system, so it is necessary to determine whether to continue training. The pseudo-positioning value predicted by the neural network is compared with the actual GNSS output position value to determine whether the error is within the expected threshold. If the error is lower than the threshold, the INS/GNSS integrated navigation is continued; otherwise, the neural network will continue to be trained while the combined navigation is performed.

### 4.3. Intelligent Integrated Navigation Algorithm

**Figure 9:**
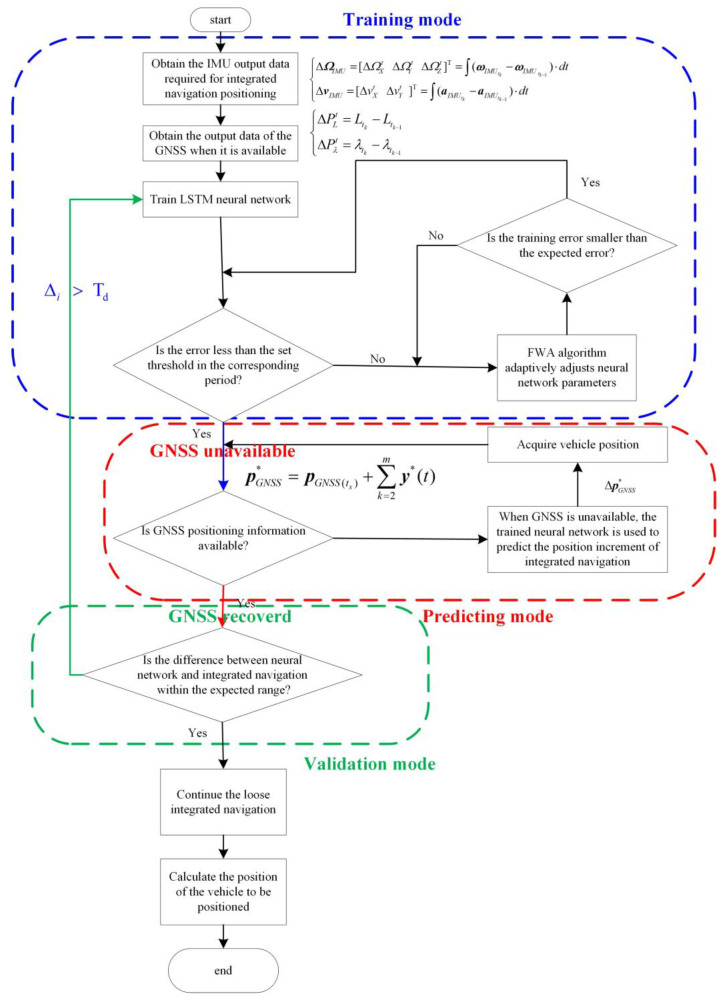
Flow chart of LSTM assisted navigation method.

Figure 9 is the flow chart of LSTM neural network-assisted navigation in this paper, which includes three parts: training mode, predicting mode, and validation mode, as well as the transformation relationship between these three parts.

Firstly, the system is in training mode. To obtain the output measurement data of the gyroscope and accelerometer required for aircraft positioning, the output data of the gyroscope and accelerometer were calculated and converted to obtain the carrier angular velocity and carrier acceleration, and store the carrier angular velocity information $\omega_{IMU}$ and carrier acceleration information $a_{IMU}$ output by the inertial navigation system during the flight of the aircraft. According to Equation (16), the angle and velocity information output by the inertial navigation system from time $t_{k-1}$ to time $t_{k}$ are differentiated to obtain the angle increment and velocity increment output by the inertial navigation system. The longitude position L and latitude position λ of the aircraft measured by the auxiliary sensor are obtained. According to Equation (18), the difference of the position information output by GNSS between time $t_{k-1}$ and $t_{k}$ is made to obtain the longitude and latitude increment of the aircraft. When GNSS is available, the output angle and velocity increment data output by INS are used for normalization processing to obtain the standardized training input sequence in Equation (17). The longitude and latitude position increment data output by GNSS is used for normalization processing to obtain the standardized training output sequence in Equation (19). The LSTM network training framework is established as described in Section 3.2, including the input layer, hidden layer, and output layer. The FWA optimization method described in Section 3.3 is used to optimize the LSTM network’s hyperparameters, including the number of hidden layers and learning rate. The training is completed until the training verification error is less than the expected error.

When GNSS fails, the system enters predictive mode. When GNSS is unavailable, the angle and velocity increment in Equation (17) of INS output after processing is transferred to the trained LSTM network for learning. The prediction array of the LSTM network shown in Equation (28) is output and de-normalized to obtain the pseudo-GNSS position increment array of LSTM network prediction. Then, the pseudo-GNSS position information can be obtained by Equation (29).

After the GNSS signal is recovered, the system enters the validation mode. Taking the GNSS positioning result as the benchmark, the system makes a difference with the pseudo-GNSS position information predicted by the LSTM network according to Equation (30) to judge whether the prediction error is within the expected error range. If yes, the training will be stopped; otherwise, the system will enter training mode, and the LSTM network will be trained again.

## 5. Simulation and Analysis

### 5.1. Simulation Setup

To improve the training speed and prediction accuracy of the LSTM network, this paper carries on the normalization processing and the incremental processing mentioned in Section 3 of the data input LSTM network.

Since the dimensions of data such as position, velocity, and angle are different, the input and output sample data can be normalized to reduce the impact of data of different dimensions on the LSTM network operation speed. Therefore, in this paper, the incremental processing results of position, velocity, and angle data are scaled to [−1, 1], respectively. Moreover, to use the LSTM predicting results to be the feedback correction of INS solution results, it is also necessary to use LSTM prediction output for the reverse normalization process.

In addition, the output of the LSTM network is the pseudo-positioning increment, so its output frequency is consistent with the output frequency of GNSS.

### 5.2. Analysis of Training Samples Influence

**Figure 10:**
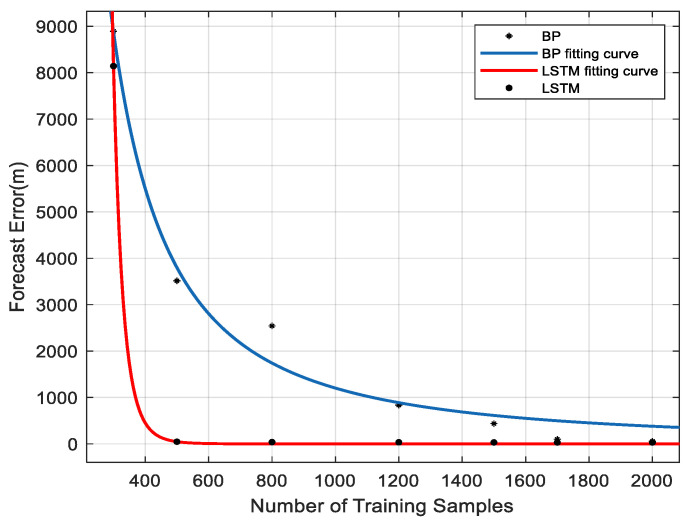
Effect of training sample number on training results.

Figure 10 shows the effect of the training sample number on training results. When the number of training samples is the same, the prediction error of LSTM is smaller than that of the BP network. When the prediction errors reached are similar, the number of training samples required by the LSTM network is less than that of the BP network, so LSTM can achieve better prediction accuracy under the condition of small samples.

**Figure 11:**
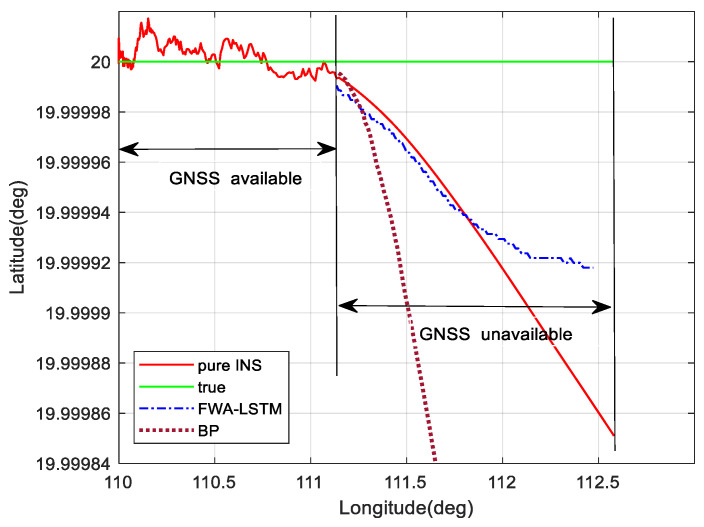
Comparison of prediction performance in small sample conditions.

To prove the performance superiority of the FWA-LSTM intelligent navigation method proposed in this paper under the condition of small samples, a simple trace simulation was carried out. The trace shown in Figure 11 is a trajectory with constant acceleration and direction. Assuming that GNSS is effective in 0–200 s and ineffective in 200–300 s, 200 groups of training samples are provided to the neural network. The FWA–LSTM prediction results proposed in this paper are compared with BP prediction results and pure inertial navigation calculation results. According to the simulation results, when the training samples are small, the network cannot be adequately trained, and the failure period is long. The LSTM-FWA network proposed in this paper can still provide the location prediction results superior to the pure inertial navigation calculation, while the prediction results of the traditional BP neural network are worse than that of the pure inertial navigation calculation. It can be seen that the intelligent navigation algorithm proposed in this paper still has better predictive performance when the number of training samples is small, and the training is not sufficient.

### 5.3. Verification of Validation Mode of Virtual Sensor

**Figure 12:**
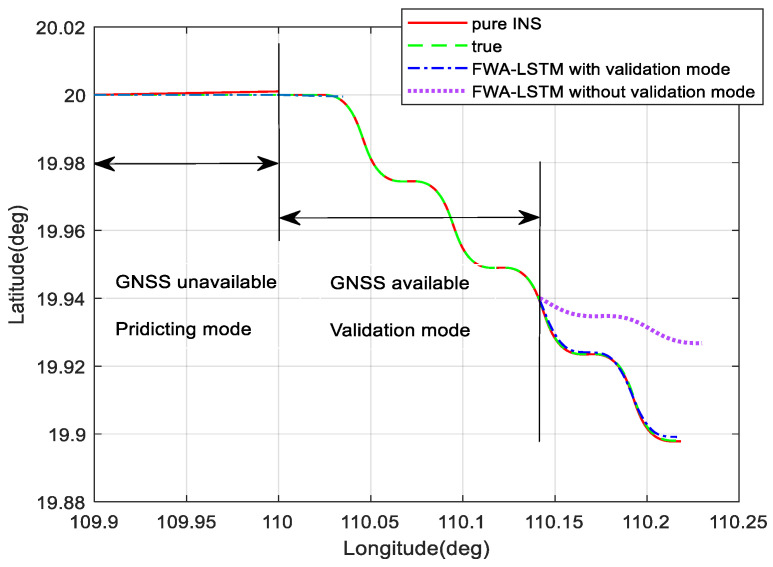
Comparison of prediction results with or without validation mode.

The trace was designed to prove the validity of the validation mode proposed in this paper, the trace was designed. When GNSS was unavailable, the system was accelerated uniformly and it was in the predicting mode. Moreover, the system entered the validation mode after the recovery of GNSS. The carrier made a curved motion, and the motion state was inconsistent with that of the previous training. As shown in Figure 12, after entering the validation mode, the prediction effect of the FWA-LSTM network in this paper cannot meet the requirements of navigation accuracy after the carrier manoeuvres. Therefore, the FWA-LSTM network will be trained in the validation mode. When GNSS fails again, it can be seen from the figure that the prediction performance of the FWA-LSTM network with validation mode is obviously better than that without validation mode.

### 5.4. Navigation Performance Analysis in GNSS Restricted Environment

**Figure 13:**
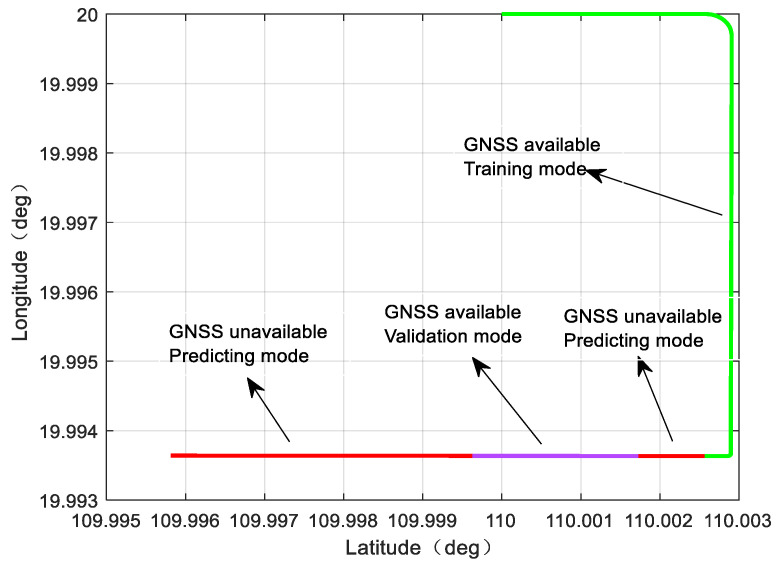
The trajectory of simulation and corresponding modes of virtual sensor.

Since the aircraft altitude information is provided by the barometric altimeter, the neural network training output information provided by the aircraft sensor is the longitude and latitude position increment. The trace image is shown as a two-dimensional image, and the flight time of the aircraft is 1500 s. Considering the GNSS failure of 900~1000 s and 1200~1500 s, the trace image is shown in Figure 13:

In the above figure, the trace image is divided into four segments. The first segment, 0~1000 s, is the GNSS available phase, marked as green, and this segment of data is used as the training input and training output of the neural network. The second segment, 900~1000 s, is the GNSS unavailable phase, marked as red, and this segment of data is used as the prediction input of the neural network. The third segment, 1000~1200 s, is the GNSS available phase, which is used to verify the network training effect and judge whether to continue training, marked as purple, and if it is necessary to continue the training, this segment of data is the training input and training output of the neural network. The fourth segment, 1200~1500 s, is the GNSS available phase, marked in red, and this segment of data is used as the prediction input of the neural network.

**Figure 14:**
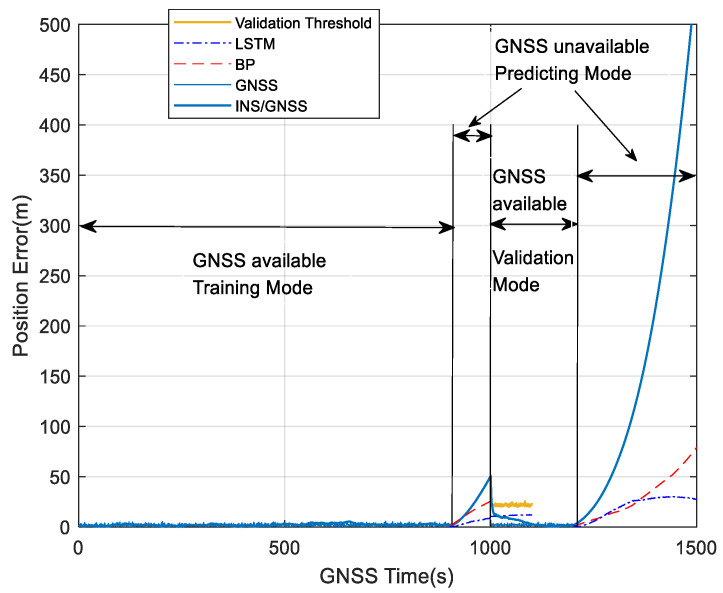
Position errors with different algorithms.

The sampling frequency of INS and GNSS is 50 Hz and 1 Hz, respectively. Figure 14 shows the navigation position error image of the whole process, which is divided into four parts, corresponding to the training path, predicting path, and validation path in Figure 13.

**Figure 15:**
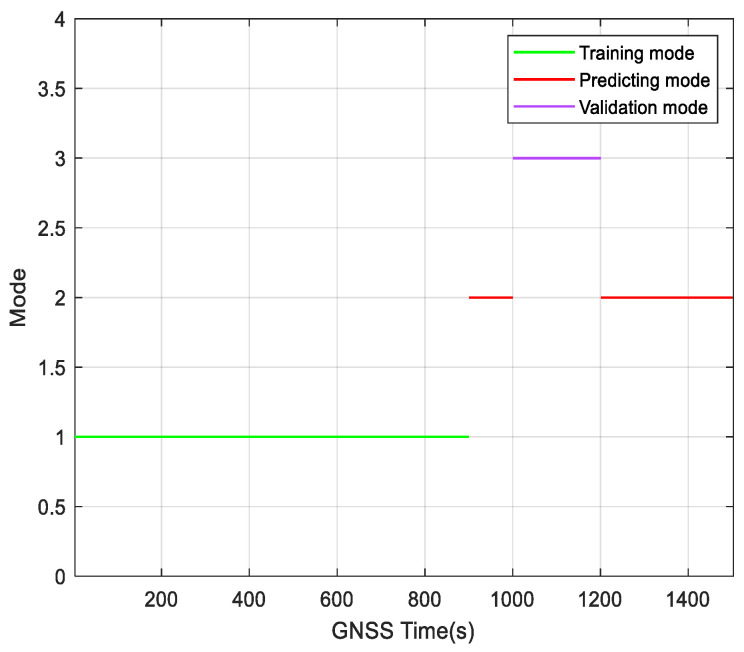
Transitions between modes of the virtual sensor.

Figure 15 shows the image of the three modes in this paper switched with the change of navigation state in the navigation process.

**Figure 16:**
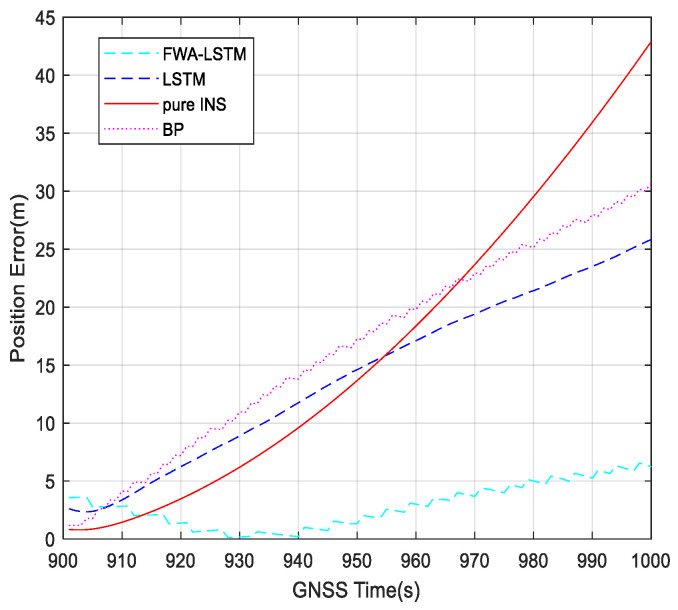
Position errors during 900~1000 s with different algorithms.

Figure 16 shows position errors during 900~1000 s with different algorithms. In 0~900 s, the GNSS receiver works normally, and the navigation system uses the loose combination to navigate. For 900~1000 s, when GNSS is unavailable, the navigation errors of four navigation modes, pure INS, BP, LSTM, and FWA-LSTM, are analyzed. The figure for position errors is shown as follows:

In Figure 16, the navigation effect of the LSTM algorithm proposed in this paper is obviously better than that of pure inertial navigation and BP neural network. The FWA-LSTM network, which is optimized by FWA for neural network parameters, also has a better prediction effect than the LSTM network with manually adjusted parameters. In Figure 11, the maximum position errors of pure INS, BP, LSTM and FWA-LSTM are 42.18 m, 29.97 m, 25.53 m, and 6.32 m, respectively.

**Figure 17:**
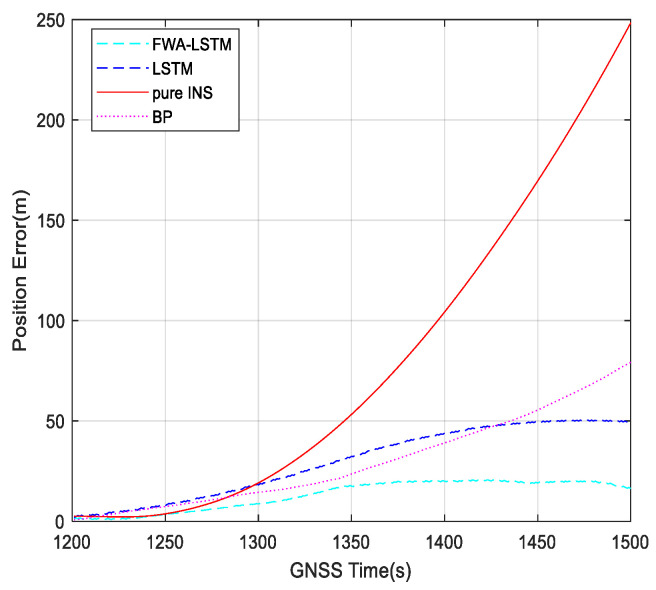
Position errors during 1200~1500 s with different algorithms.

Figure 17 shows the position errors during the 1200~1500 s with different algorithms. Within 1000~1200 s, the GNSS signal is available, and the navigation system uses the loose combination to navigate. Based on the recovered GNSS navigation position results, the position error between the predicted value and the true value of the neural network is 6.5 m, as is shown in Figure 14, which is within the range of the set threshold of 20 m. Therefore, it is not necessary to continue to train the LSTM network and save the system running time. For 1200~1500 s, when GNSS is unavailable, the navigation errors of four navigation modes, pure INS, BP, LSTM, and FWA-LSTM, are analyzed. The figure for position errors is shown as follows:

In Figure 17, the navigation effect of the LSTM algorithm proposed in this paper is obviously better than that of pure inertial navigation and BP neural network. The FWA-LSTM network, which is optimized by FWA for neural network parameters, also has a better prediction effect than the LSTM network with manually adjusted parameters. In Figure 17, the maximum position errors of pure INS, BP, LSTM, and FWA-LSTM are 247.53 m, 77.7 m, 48.84 m, and 15.54 m, respectively. All the results are summarized in Table 1.

When GNSS signals are available, the system is in training mode. The angle and velocity increment measured by INS is used as the training input of the neural network, while the position increment measured by GNSS is used as the training output. When the GNSS signal is unavailable, the whole system moves into predicting mode. The angle and velocity increment measured by INS is used as the predictive input of the neural network, and the predictive output of the LSTM network is the position increment. Then, by accumulating these position increments, the pseudo positioning can be obtained to replace the real position measured by GNSS. The difference between the pseudo positioning and the position of INS constitutes a part of the input vector of the Kalman filter. Finally, the Kalman filter is used to correct the errors of the inertial navigation system and reduce the divergence of the errors. The simulation results in Table 1 show that the BP algorithm cannot be used as an appropriate model to assist INS during the interruption of the GNSS signal because it cannot deal with the time dependence between the current and past motion states. When GNSS is unavailable, the LSTM algorithm can construct the relationship between future and past information and obtain more accurate navigation results.

Furthermore, as the inertial navigation output frequency can reach hundreds of Hertz, it can meet the requirements of a high dynamic carrier, and its accuracy can be maintained by regular correction through the Kalman filter. In the predicting mode, the running time of the program for one prediction is 0.015943 s, which is within one Kalman filtering cycle (1 s). Therefore, the algorithm time can be controlled within one filtering cycle. It can be concluded that the fully trained LSTM network can predict the location of incremental information in a short time and meet the needs of practical applications.

## 6. Conclusions

In this paper, an intelligent navigation method using LSTM neural network-assisted INS/GNSS loose combination is proposed. By predicting the position increment under the condition of GNSS failure, the pseudo-GNSS position is obtained, and the INS is combined with the Kalman filter to reduce the divergence error of inertial navigation. The prediction effect of the LSTM network is compared with that of the BP network. Since the BP network cannot process time-related sequences, the prediction effect is worse than that of the LSTM network. However, the LSTM network proposed in this paper can process time-related sequences well and transform among training mode, predicting mode and verification mode, making it more flexible and more accurate in prediction effect. At the same time, this paper also proposes a parameter optimization method for LSTM based on the FWA algorithm, which avoids the problem of low efficiency and the general prediction effect of traditional manual parameter optimization for LSTM. Simulation results show that the LSTM network with FWA parameter optimization has higher prediction accuracy. Therefore, the LSTM neural network-assisted INS/GNSS intelligent navigation method proposed in this paper can provide effective help to suppress inertial dispersion during the period when GNSS is unavailable.

## Figures and Tables

**Table 1:** Max position error during GNSS unavailable period.

		Pure INS	BP	LSTM	FWA-LSTM
Max position error (m)	900~1000 s	42.18	29.97	25.53	6.24
	1200~1500 s	247.53	77.7	48.84	16.51

## Data Availability

The data presented in this study are available on request from the corresponding author.
